# First Trimester Aneuploidy Screening Markers in Women with Pre-Gestational Diabetes Mellitus

**DOI:** 10.3390/jcm3020480

**Published:** 2014-05-08

**Authors:** Padmalatha Gurram, Peter Benn, James Grady, Anne-Marie Prabulos, Winston Campbell

**Affiliations:** 1 Department of Obstetrics and Gynecology, University of Connecticut Health Center-School of Medicine, Farmington, CT 06032, USA; E-Mails: prabulos@up.uchc.edu (A.-M.P.); wcampbell@nso2.uchc.edu (W.C.); 2 Department of Genetics and Developmental Biology, University of Connecticut Health Center-School of Medicine, Farmington, CT 06032, USA; E-Mail: benn@nso1.uchc.edu; 3 Biostatistics Center, Connecticut Institute for Clinical and Translational Science, University of Connecticut Health Center-School of Medicine, Farmington, CT 06032, USA; E-Mail: jgrady@uchc.edu

**Keywords:** pre-gestational diabetes, aneuploidy, first trimester screening, PAPP-A, β-hCG, correction factors, HbA1c

## Abstract

**Objective:** To investigate whether maternal serum pregnancy associated plasma protein-A (PAPP-A), total β human chorionic gonadotropin (hCG) levels and nuchal translucency (NT) measurements differ in women with pre-gestational diabetes mellitus (PGDM) compared to non-diabetic controls and to assess whether correction factors are needed for diabetic women in calculation of aneuploidy risks. **Study Design:** We performed a retrospective study of all women who underwent first trimester aneuploidy screening (11 + 0 to 13 + 6 weeks) from 2005 to 2011. The primary study outcome was the difference in PAPP-A, β-hCG and NT multiples of median between women with PGDM and non-diabetic women. **Results:** Of 6741 eligible patients, 103 patients with PGDM were using insulin and 4 patients were using oral hypoglycemic agents; the latter were excluded due to small number. There was 12% reduction of median PAPP-A (*p* = 0.001) and 18% reduction of median hCG (*p* = 0.006) in women with PGDM receiving insulin. There was no difference in NT. **Conclusions:** In women with PGDM receiving insulin, PAPP-A and β-hCG levels are significantly lower compared to non-diabetic women. This suggests that when calculating risks for aneuploidy, correction factors should be considered to adjust PAPP-A and β-hCG concentrations to those seen in non-diabetic women.

## 1. Introduction

Maternal factors such as weight, race, parity, multiple gestation, smoking, *in vitro* fertilization and diabetes mellitus are known to affect the detection rates and false positive rates of the screening tests for autosomal trisomies [1,2]. The first serum analyte used to screen for birth defects was maternal serum alpha-fetoprotein (MSAFP). Elevated MSAFP values could identify pregnancies at risk for open neural tube defects [3]. Subsequently, it was reported low MSAFP values are useful as a screen for pregnancies at risk of fetal Down syndrome [4].

In women with pre-gestational diabetes (PGDM), the second trimester MSAFP is decreased. Unless a correction factor is applied, this falsely increases the calculated risk for Down’s syndrome [5]. Most laboratories use a 10% to 20% upward adjustment to MSAFP in their second-trimester screening tests for aneuploidy for women with insulin dependent PGDM [6]. However, there is considerable variation in the literature regarding first trimester serum screening analyte values in women with PGDM.

First trimester screening for aneuploidy (trisomies 21 and 18) includes measurement of maternal serum pregnancy associated plasma protein-A (PAPP-A), beta human chorionic gonadotropin (hCG, either free or total) and ultrasound measurement of fetal nuchal translucency (NT, the fluid filled space behind the fetal neck) at 11 + 0 to 13 + 6 weeks of gestation with results combined with maternal age to compute aneuploidy risk. The first trimester screening test has a reported detection rate of approximately 80%–87% using a fixed false positive rate of 5% for Down syndrome [7].

It was initially reported that there was no difference in PAPP-A, free β-hCG or NT between PGDM women and non-diabetic controls [8]. More recent studies have shown that median PAPPA levels [9,10,11] and β-hCG levels are decreased in women with pre-gestational diabetes [10,11]. Nix *et al*. reported that a 10% bias in a component marker MoM (multiple of median) values can change the false positive rate of the screening test by up to 2% [12]. PAPP-A expression has been shown to be inversely related to glycosylated hemoglobin (HbA1c) in non-pregnant diabetic women which could explain low PAPP-A levels in diabetic pregnancies [13,14]. Reports on first trimester hCG concentrations have shown either normal or reduced levels in diabetic women [8,9,10,11,14]. Based on these observations, we hypothesize that first trimester screening markers are different in pre-gestational diabetics *versus* non-diabetics.

The objective of this study was to investigate if the maternal serum PAPP-A, total β-hCG levels and fetal NT measurements differ in women with PGDM compared to the non-diabetic controls and whether correction factors might be needed to adjust the levels to non-diabetic levels. In addition, we aimed to compare the false positive screening rates between the PGDM and non-diabetics, and evaluate whether there is any correlation between the first trimester aneuploidy markers and HbA1c levels.

## 2. Material and Methods

### 2.1. Study Design

This is a retrospective cohort analysis of all pregnant women who underwent first trimester screening for trisomies 21 and 18 at the University of Connecticut Health Center from January 2005 to December 2011. Women received their routine obstetric care from community physicians and were referred to the University for first trimester ultrasound exams with the biochemical testing carried out in our regional screening laboratory. This aneuploidy screening was performed using a combination of ultrasound measurement of fetal nuchal translucency (NT) and maternal serum analyte markers total β-hCG and PAPP-A.

Gestational age was determined according to crown-rump length (CRL) measurements. Fetal NT and CRL were measured by standardized techniques by Nuchal Translucency Quality Review (NTQR) certified sonographers. All images were reviewed by the NTQR certified Maternal-Fetal Medicine specialists. Maternal serum PAPP-A and total β-hCG were measured in the laboratories at the University of Connecticut Health Center. The serum analyte tests were carried out on fresh samples with quality control metrics based on widely accepted standards [15].

The inclusion criteria were women with singleton pregnancies who underwent screening for trisomies 21 and 18 between 11 + 0 and 13 + 6 weeks of gestation in our prenatal ultrasound unit. Women who underwent invasive testing and were diagnosed with a fetal chromosomal abnormality or who were known to have a full term aneuploid pregnancy were excluded. Patients who were known to have developed gestational diabetes and delivered at our institution were also excluded because of uncertainty about the time of diagnosis and questionable carbohydrate intolerance at the time of aneuploidy screening. Women were classified as having pre-gestational diabetes mellitus, if they had been using insulin or oral hypoglycemic agents prior to pregnancy. No distinction was made between pre-gestational type-1 and type-2 diabetes.

### 2.2. Data Collection

Maternal demographic characteristics, ultrasound findings recorded at the time of screening, results of biochemical testing and data on pregnancy outcome were obtained from our computerized databases. The patient’s measurement of HbA1c was accepted for inclusion in the study if the blood sample was performed at University of Connecticut Health Center in a time period from 8 weeks before to 8 weeks after the first trimester aneuploidy screening blood draw. The study was approved by the University of Connecticut Health Center Institutional Review Board.

### 2.3. Outcome Measures

The primary outcome measure was the difference in the multiples of median of the distribution of serum biochemical markers (PAPP-A, total β-hCG) and NT between the women with pre-gestational diabetes and non-diabetic patients. The laboratory database contained the concentrations of the biochemical markers expressed as gestational age adjusted, weight, and race/ethnicity corrected multiples of median (MoMs) [16]. NT was similarly expressed as gestational age adjusted MoMs.

The secondary outcomes of the study included comparing the Down syndrome screen positive rates for women with pre-gestational diabetes to non-diabetic women, determining the relationship between first trimester serum screening analytes (PAPP-A, hCG) and maternal HbA1c levels. We also examined if the effect of PGDM on first trimester analytes was dependent on gestational age.

### 2.4. Statistical Methods

In a study of 489 pregnancies by Spencer *et al*. [9], levels of PAPP-A were significantly reduced by 15%. Performing a power analysis based on these results, for an effect size (Cohen) of 0.5, a sample size of 64 in each group would have 80% power to detect a difference in means with a two-tailed alpha of 0.05 [17].

Analyses were performed using SPSS-20 software (IBM Corp., Armonk, NY, USA). The Kolmogorov-Smirnov test was used to check for the normality of data distribution. Chi-square test (or Fishers exact test, if the table cell sizes are small and warrant exact methods) was used to compare rates between groups for categorical outcome variables. Comparisons between groups for continuous outcome variables were assessed using an independent *t*-test. For non-parametric data, Mann-Whitney *U*-Test and Kruskal-Wallis tests were used. All tests used a two sided alpha level of significance of 0.05.

## 3. Results

### 3.1. Study Sample

**Figure 1:**
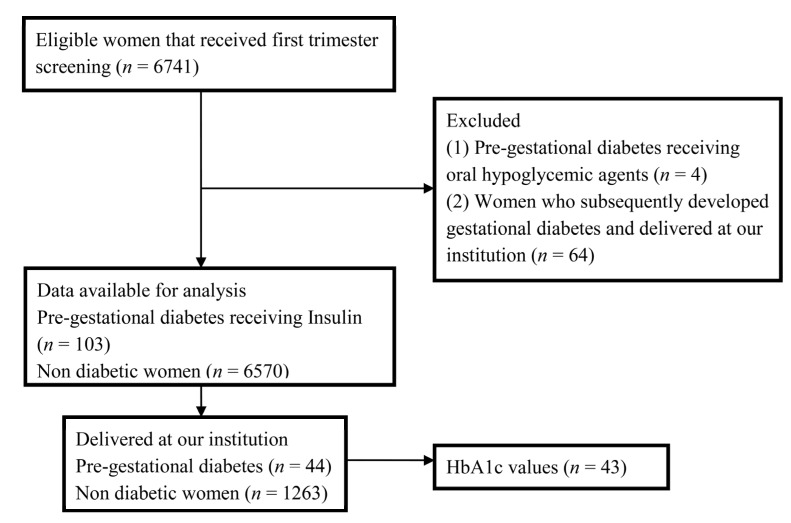
Flow chart of studypopulation.

Between 2005 and 2011, there were 6741 eligible patients who underwent first trimester screening. Of the eligible patients, 103 (1.5%) women with pre-gestational diabetes were receiving insulin prior to the pregnancy (PGDM) and 4 patients were receiving oral medications. Due to the small size of the sample, patients who were using oral hypoglycemic agents were excluded from further analysis. Of the 6737 patients, 1327 patients were delivered at University of Connecticut Health Center. Gestational diabetes mellitus was diagnosed in 64 women (4.8%) who delivered at University of Connecticut Health Center, and these patients were also excluded leaving 6673 for final analysis (Figure 1).

### 3.2. Maternal Characteristics

Maternal characteristics are shown in Table 1. There was no significant difference in the maternal age and gravidity between the groups. Women with PGDM weighed on average 41 lbs more than those with non-diabetic pregnancies (*p* < 0.001). Patients with PGDM were more likely to be of Black race (19%) compared to non-diabetics (7.5%, *p* < 0.001). There was no significant difference between PGDM and non-diabetics in gestational age and crown-rump length (CRL) at the time of first trimester screening.

**Table 1:** Descriptive demographics of study patients.

Variables	PGDM ^† ^(*n* = 103)	Non-Diabetic (*n* = 6570)	*p* Value
Maternal age ^∞^, years, mean ± SD	31.8 ± 6.1	31.5 ± 5.9	0.60
Weight ^μ^, lbs, mean ± SD	202.3 ± 59.1	161.6 ± 39.2	<0.001
Gestational age at screening, weeks, mean ± SD	12.5 ± 0.6	12.6 ± 0.5	0.23
CRL at screening, mm, mean ± SD	63.6 ± 8.7	64.4 ± 7.8	0.30
Race/ethnicity ^∆,^* *n*	84	5340	<0.001
White, *n* (%)	50 (59.5)	4042 (75.7)	
Black, *n* (%)	16 (19.0)	399 (7.5)	
Hispanic, *n* (%)	11 (13.1)	572 (10.7)	
Others, *n* (%)	7 (8.3)	327 (6.1)	
Gravidity ^∆, €^, *n*	42	1219	0.23
Primigravida, *n* (%)	9 (21.4)	366 (30.0)	
Multigravida, *n* (%)	33 (78.6)	853 (70.0)	

^†^ Pre-gestational diabetes mellitus receiving insulin; ^∞^ Maternal age calculated at estimated date of delivery; ^μ^ Maternal weight determined at the time of first trimester screening. Analysis by Student’s *t* test for continuous variables and *x*^2^ (^∆^) for categorical variables; * The data for race is available for only 5424 patients; ^€^ The data for gravidity is available for only 1241 patients.

### 3.3. Maternal Serum PAPP-A, Total Beta HCG and Fetal NT

There was 12% reduction in median PAPP-A (*p* = 0.001) and 18% reduction in total hCG in women with PGDM (*p* = 0.006) (Table 2). There was no significant difference in NT between PGDM and non-diabetic patients (*p* = 0.35).

**Table 2:** Comparison of first trimester analytes between PGDM and non-diabetics.

Variables	PGDM ^† ^(*n* = 103)	Non-Diabetic (*n* = 6570)	*p* Value
PAPP-A * MoM ^μ^	0.86 (0.51–1.22)	0.98 (0.69–1.39)	0.001
Total beta hCG ^€^ MoM	0.83 (0.68–1.22)	1.01 (0.74–1.34)	0.006
NT ^£^ MoM	1.09 (0.94–1.27)	1.08 (0.93–1.2)	0.35

^†^ Pre-gestational diabetes mellitus receiving insulin; ^μ^ Multiples of the Median; * Pregnancy associated plasma protein; ^€^ Human chorionc gonadotropin; ^£^ Nuchal translucency. Data are median (Interquartile range). Statistical analysis performed using the Mann-Whitney *U* test.

### 3.4. Screening Test Performance

The false positive rates for Down’s syndrome at risk cut-offs 1:270, 1:150, 1:100 and 1:50 were compared between the groups using Fisher’s exact test (Table 3). The false positive rates were higher in the PGDM group and the difference was most evident for those with risks 1:50 or greater (*p* = 0.04).

**Table 3:** Proportion of pregnancies with estimated risk for Down syndrome above cut-offs of 1:270, 1:150, 1:100 and 1:50 among the groups.

Outcome Groups	Risk ≥ 1:270	Risk ≥ 1:150	Risk ≥ 1:100	Risk ≥ 1:50
PGDM ^†^, (*n* = 103), *n* (%)	9 (8.74)	8 (7.77)	5 (4.85)	4 (3.88)
Non-diabetic, (*n* = 6570), *n* (%)	472 (7.18)	258 (3.93)	171 (2.60)	80 (1.22)
*p*-Value	0.56	0.07	0.20	0.04

^†^ Pre-gestational diabetes mellitus receiving insulin. Statistical analysis performed using Fisher’s exact test.

### 3.5. Glycemic Control and First Trimester Screening Markers

**Figure 2:**
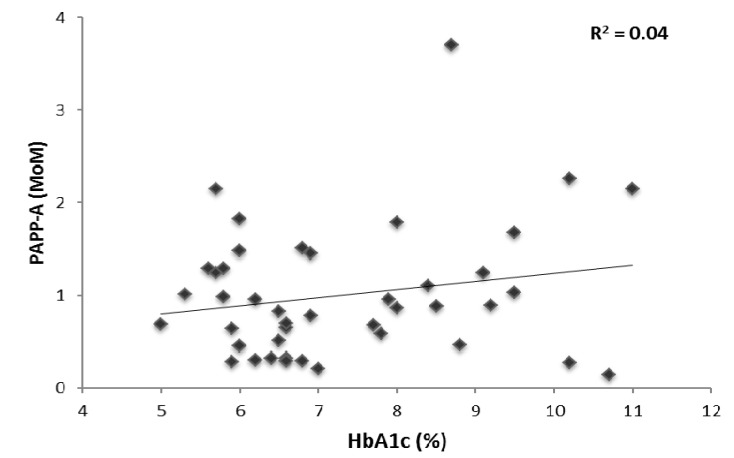
Scatter plot showing the correlation between pregnancy associated plasma protein-A (PAPP-A) multiples of median (MoM) and glycosylated hemoglobin percent (%).

There were 103 patients with PGDM. Of these 44 delivered at our institution and 43 had HbA1c values measured within 8 weeks of serum screening. The mean HbA1c was 7.3 ± 1.5. The Pearson correlation coefficient showed there was no statistically significant correlation between HbA1c and PAPP-A (*r* = 0.20, *p* = 0.19, Figure 2), totalHCG (*r* = −0.05, *p* = 0.74) or NT(*r* = 0.05, *p* = 0.70).

### 3.6. Gestational Age and First Trimester Screening Markers in PGDM

The serum PAPP-A, total beta hCG concentration and NT measurement (expressed as MoMs) obtained in patients at 11–11.6 weeks gestation was compared to values obtained in patients at 12–12.6 weeks or 13–13.6 weeks gestation for the insulin dependent PGDM women (Table 4). PAPP-A levels appeared to increase with gestational age but the trend was not statistically significant (*p* = 0.30). No clear trend was noted between either total hCG or NT and gestational age.

**Table 4:** Gestational age related effects of PGDM ^†^ on first trimester screening analytes.

Variables	11–11.6 Weeks (*n* = 18)	12–12.6 Weeks (*n* = 60)	13–13.6 Weeks (*n* = 25)	*p* Value
PAPP-A * MoM ^μ^	0.69 (0.44–1.51)	0.78 (0.44–1.17)	0.95 (0.71–1.26)	0.30
Total hCG ^€^ MoM	0.78 (0.57–0.87)	0.88 (0.68–1.22)	0.83 (0.74–1.41)	0.22
NT ^£^ MoM	1.05 (0.82–1.32)	1.12 (0.98–1.3)	1.02 (0.90–1.21)	0.18

^†^ Pre-gestational diabetes mellitus receiving insulin; ^μ^ Multiples of the Median; * Pregnancy associated plasma protein; ^€^ Human chorionc gonadotropin; ^£^ Nuchal translucency; Data are median (Interquartile range); Statistical analysis performed using the Kruskal-Wallis test.

## 4. Discussion

Our findings show that the levels of maternal serum analytes, PAPP-A and total beta hCG used in first trimester aneuploidy screening are decreased in women with PGDM receiving insulin. Furthermore, the false positive rates of first trimester screening are higher in pregnancies with PGDM compared to non-diabetic women when these differences are not taken into consideration.

In our study sample of women with PGDM receiving insulin, the levels of PAPP-A were significantly reduced by 12%. This is consistent with several published studies that reported 5%–20% reduction in the PAPP-A in women with unspecified types of diabetes mellitus [8,9,14]. Our study did not differentiate between Type-1 and Type-2 diabetes mellitus. A previous study by Madison *et al.* showed a significant reduction in PAPP-A levels in women with Type-1 diabetes mellitus (0.86 compared to 1.01 MoM in non-diabetics, *p* < 0.001) [14]. A study by Savvidou *et al.* showed that PAPP-A was reduced by 25% in Type-2 diabetes and there was a 9% (non-significant) reduction in Type-1 diabetes at 11–13 weeks of gestation [10]. Due to the small sample size of our study, we were unable to study the impact of diabetic women receiving oral hypoglycemic agents on first trimester screening.

In our study sample the level of total hCG was significantly reduced by 18% in women with PGDM. This is in contrast with most other studies which reported no difference in free β-hCG in women with pre-gestational diabetes [8,9,10,14]. Only one study showed a reduction in free β-hCG [11]. We are aware of only one study that reported the effect of pre-gestational diabetes on total hCG. That study found that total hCG is not altered in women with pre-gestational diabetes [2]. We found that fetal nuchal translucency measurements were similar in PGDM and non-diabetics; these results are consistent with other studies [8,9,14].

Our study showed that the false positive screening tests for Down syndrome are increased in women with PGDM using insulin. This is consistent with the findings of Ball *et al.* [11]. Our results are also similar to those of Savvidou *et al.* who reported the false positive rates of first trimester screening doubled in women with Type-2 diabetes [10].

In pregnancies with Down Syndrome, serum PAPP-A levels are decreased and β-hCG levels are increased. If both PAPP-A and β-hCG are lowered, it is expected that they counteract each other and the false positive rates for Down syndrome remain unchanged. The finding of an increased false positive rate may indicate that, with similar lowering of the two markers, PAPP-A outweighs total β-hCG. In pregnancies with trisomy 18, both PAPP-A and beta hCG are decreased. The false positive rates for trisomy 18 are expected to be increased, if both the markers are decreased. However, due to small number of patients we were not able to compare the false positive rates for trisomy 18 in our study.

Although the PAPP-A MoMs in PGDM women appeared to increase markedly with increasing gestational age, the trend did not achieve statistical significance. No significant trends were seen with β-hCG and NT in relation to gestational age in our study sample. These observations are very similar to that of Ball *et al.* [11] who also reported non-significant trends in PAPP-A, free β-hCG and NT with gestational age. They also noted that although the analytes, separately showed non-significant gestational age dependencies, a failure to adjust for PAPP-A and β-hCG would result in an increase of false positive results. They estimated that for the combined test with free β-hCG, the likelihood ratio was altered by a factor of 3.6 at 9 weeks and 1.3 at 13 weeks in women with Type-2 diabetes. This translated into an increase in false positive rates of about 90% at 9 weeks and 21% at 13 weeks [11].

Previous studies in non-pregnant diabetics showed an inverse relationship between HbA1c (marker of glycemic control) and PAPP-A suggesting that PAPP-A levels are related to glycemic control [13]. It is possible that PGDM with normal HbA1c might have normal PAPP-A levels and only poorly controlled diabetics might need correction. Our study did not show any significant correlation between first trimester screening analytes and HbA1c. However, our study is limited in terms of number of patients with known HbA1c values around the time of first trimester screening. HbA1c is generally considered to be a good reflection of glucose control in diabetic patients but a limitation of our study is that this testing was not carried out at the time of the hCG and PAPPA measurements. Studies with more patients are needed to further evaluate these findings.

PAPP-A is a member of metzincen superfamily of metalloproteinases that regulates extracellular matrix remodeling [18]. In non-pregnant individuals, it is produced by human fibroblasts, osteoblasts and vascular smooth muscle [19]. PAPP-A regulates insulin-like growth factor axis (IGF axis) and has shown to be involved with glycemic control and atherosclerosis [10,20,21]. PAPP-A is expressed in both eroded and ruptured atherosclerotic plaques and has been shown to be a marker of early diagnosis of acute coronary syndromes [18,21]. Our study is not designed to answer questions such as whether PAPP-A levels are decreased only in pre-gestational diabetic women with vascular disease. Additional studies are required to elucidate the reason for low PAPP-A in diabetic women and to assess the relationship between the PAPP-A levels and PGDM women with vascular disease.

Previous studies have shown varying results regarding the PAPP-A levels in patients who subsequently developed gestational diabetes [10,22]. In the study by Beneventi *et al.*, PAPPA levels were significantly lower in women who subsequently developed gestational diabetes (1.2 MoM *vs.* 0.7 MoM, *p* < 0.005), suggesting that PAPP-A could be a marker for glucose intolerance [22]. However, the study by Savvidou *et al.* did not find any significant difference in PAPP-A levels in the patients who subsequently developed gestational diabetes mellitus [10]. In our study, we were only able to identify patients who developed gestational diabetes if they delivered at our institution.

The strengths of our study include using data from one institution where all aneuploidy markers were analyzed in one lab and included an analysis of the effects of PGDM on total hCG. The limitations of our study includes the small sample size of the patients with values of HbA1c results, not being able to distinguish between Type 1 and Type-2 diabetes mellitus and not being able to separately assess the group of PGDM women using oral hypoglycemic agents.

## 5. Conclusions

We conclude that PGDM women receiving insulin, correction factors should be considered to adjust the maternal serum PAPP-A and total β-hCG concentrations to those seen in non-diabetic women. Additional studies are required to determine the amount of adjustment and the overall effect of these adjustments on the performance of first trimester aneuploidy screening tests.
